# Application of TiO_2_ Supported on Nickel Foam for Limitation of NO_x_ in the Air via Photocatalytic Processes

**DOI:** 10.3390/molecules29081766

**Published:** 2024-04-12

**Authors:** Beata Tryba, Bartłomiej Prowans, Rafał Jan Wróbel, Paulina Szołdra, Waldemar Pichór

**Affiliations:** 1 Department of Catalytic and Sorbent Materials Engineering, Faculty of Chemical Technology and Engineering, West Pomeranian University of Technology in Szczecin, Pułaskiego 10, 70-322 Szczecin, Poland; bartlomiej.prowans@zut.edu.pl (B.P.); rafal.wrobel@zut.edu.pl (R.J.W.); 2 Department of Building Materials Technology, Faculty of Materials Science and Ceramics, AGH University of Krakow, Al. Mickiewicza 30, 30-059 Kraków, Poland; szoldra@agh.edu.pl (P.S.); pichor@agh.edu.pl (W.P.)

**Keywords:** NO_x_ abatement, TiO_2_/nickel foam, NO oxidation, photocatalysis

## Abstract

TiO_2_ was loaded on the porous nickel foam from the suspended ethanol solution and used for the photocatalytic removal of NO_x_. Such prepared material was heat-treated at various temperatures (400–600 °C) to increase the adhesion of TiO_2_ with the support. Obtained TiO_2_/nickel foam samples were characterized by XRD, UV–Vis/DR, FTIR, XPS, AFM, SEM, and nitrogen adsorption at 77 K. Photocatalytic tests of NO abatement were performed in the rectangular shape quartz reactor, irradiated from the top by UV LED light with an intensity of 10 W/m^2^. For these studies, a laminar flow of NO in the air (1 ppm) was applied under a relative humidity of 50% and a temperature of 28 °C. Concentrations of both NO and NO_2_ were monitored by a chemiluminescence NO analyzer. The adsorption of nitrogen species on the TiO_2_ surface was determined by FTIR spectroscopy. Performed studies revealed that increased temperature of heat treatment improves adhesion of TiO_2_ to the nickel foam substrate, decreases surface porosity, and causes removal of hydroxyl and alcohol groups from the titania surface. The less hydroxylated surface of TiO_2_ is more vulnerable to the adsorption of NO_2_ species, whereas the presence of OH groups on TiO_2_ enhances the adsorption of nitrate ions. Adsorbed nitrate species upon UV irradiation and moisture undergo photolysis to NO_2_. As a consequence, NO_2_ is released into the atmosphere, and the efficiency of NO_x_ removal is decreasing. Photocatalytic conversion of NO to NO_2_ was higher for the sample heated at 400 °C than for that at 600 °C, although coverage of nickel foam by TiO_2_ was lower for the former one. It is stated that the presence of titania defects (Ti^3+^) at low temperatures of its heating enhances the adsorption of hydroxyl groups and the formation of hydroxyl radicals, which take part in NO oxidation. Contrary to that, the presence of titania defects in TiO_2_ through the formation of ilmenite structure (NiTiO_3_) in TiO_2_/nickel foam heated at 600 °C inhibits its photocatalytic activity. No less, the sample obtained at 600 °C indicated the highest abatement of NO_x_ due to the high and stable adsorption of NO_2_ species on its surface.

## 1. Introduction

The photocatalytic removal of nitrogen oxides on TiO_2_ represents a significant area of research in environmental protection and clean technology. Nitrogen oxides (NO_x_) are major atmospheric pollutant gases originating mainly from industrial emissions, transport, and combustion processes. Their presence in the air has harmful effects on human health and ecosystems. Therefore there are some processes applied in the industry for the limitation of nitrogen oxides to the atmosphere, such as SCR (selective catalytic reduction) or wet denitration in ammonia or urea solutions [1]. Some other processes of NO_x_ abatement have been also studied, which are carried out at low temperatures such as catalytic ozonation, electrochemical reduction by single-atom catalysts, or photo-deNOx reactions [2,3,4].

Photocatalytic removal of nitrogen oxides on TiO_2_ is one of the most promising approaches to address this problem, using advanced nanotechnology materials and photocatalytic processes [5]. TiO_2_, or titanium dioxide, has become a focus of research due to its unique photocatalytic properties. This effect is based on the ability of TiO_2_ to activate photocatalytic processes under UV or visible light, leading to the breakdown of gaseous pollutants into harmless products. However, the resulting by-products are not always completely safe and non-toxic to the environment. In the case of photocatalytic oxidation of nitric oxide, nitrogen dioxide is produced, which can adsorb on the TiO_2_ surface and be transformed to nitrogen ions but can also desorb from the surface, contributing to toxicity levels in the atmosphere. It is therefore important that the photocatalytic oxidation of NO proceeds with the lowest possible NO_2_ yield at the end of the process. Previous scientific studies have shown that the conversion of NO_2_ to NO_3_^−^ ions is faster under humid conditions. In addition, the presence of alkaline ions on the TiO_2_ surface, such as K^+^, results in the trapping of NO_3_^−^ ions and their stronger binding to the titania [6]. In the case of other technology used for NO_x_ removal such as wet denitration in the urea solution, the presence of alkali conditions allowed for dissolving urea nitrate, which was formed as a side product and being in excess was disadvantageous [1]. Another way to increase nitrogen oxide removal efficiency while reducing NO_2_ release is to modify TiO_2_ with noble metals. Recent research efforts have explored the modification of TiO_2_ photocatalysts with noble metals such as platinum (Pt), palladium (Pd), silver (Ag), and gold (Au) to enhance their photocatalytic NO_x_ removal efficiency [7,8,9]. It was proved that noble metals act as co-catalysts, promoting charge separation, as well as facilitating the adsorption and transformation of NO_x_ species. Noble metals can increase the reaction rates of NO oxidation to NO_2_ and nitrite oxidation to nitrate, resulting in an increase in NO_x_ adsorption and nitrate formation [10]. The properties of TiO_2_ material are also essential in the efficacy of NO removal. The high surface area of TiO_2_ and the presence of Ti^3+^ centers are favorable features enhancing its photocatalytic activity towards NO oxidation [11,12]. Reduced TiO_2_ by plasma treatment indicated high photocatalytic activity towards NO conversion to NO_3_^−^ under both UV and visible lights due to the formation of an oxygen vacancy state between the valence and the conduction bands in the TiO_2_ band structure, which resulted in the intensification of electron traps [11]. However, for a real application of TiO_2_ for the removal of NO_x_, it has to be immobilized on the support. Different supported materials have been already tested, such as asphalt, pavement, cement, mortar, glass, polymers, steel, nickel foam, and others [13,14,15,16,17,18]. The interaction of support with TiO_2_ occurs in the case of a thin layer coating. In the case of TiO_2_-coated cement, it was reported that the NO_x_ reduction was improved when zeolite and activated red clay were used as cement substitutes [18]. The other researchers indicated the strong and disadvantageous interaction of glass or Teflon with titania crystallites conducting to decrease its photocatalytic abilities whereas stainless or sand-blasted steel used as the supports were very attractive materials due to their conductive properties, which might in fact improve the separation of charge carriers, thus enhancing the photocatalytic process [14]. Therefore, the application of nickel foam as a support for TiO_2_ seems to be a very good solution due to its conductive properties and porous structure. In the literature, a published paper related to the application of TiO_2_-coated nickel foam for NO_x_ removal was found [17]. The authors of this paper demonstrated that good adhesion of TiO_2_ to the nickel foam occurred after heat treatment of this composite at 600 °C, they observed interdiffusion of NiO and TiO_2_, which resulted in the formation of the NiTiO_3_ phase. Such prepared composites revealed enhanced NO removal under visible light by comparison with powdered TiO_2_. These researchers also underlined that the porous structure of the nickel foam provided sufficient contact between the photocatalyst and gaseous pollutants and better utilization of incident photons. However, the adsorption of gaseous molecules can be variable not only by the porous structure of the photocatalyst but also by its chemical properties. Some researchers prepared a porous titania foam and tested it for the photocatalytic removal of NO_x_ [14]. They obtained enhanced photocatalytic yield of NO_X_ abatement when covering the titania foam surface with the amphiphilic compound (hexylic acid or hexylamine). Such modification of the titania surface caused increased adsorption of reactant gas molecules. The method of TiO_2_ coating can affect its surface properties as well. TiO_2_ in the aqueous suspension can form some agglomerates and when sprayed onto support can affect the surface roughness. Therefore, some surfactants are used to improve the dispersion of titania nanoparticles. Some researchers noted an increased photocatalytic activity of TiO_2_ coating roadside plates towards NO_x_ removal when they added a surfactant (sodium dodecylbenzene sulfonate) to the titania suspension while coating [13]. In the presented studies, anatase-type TiO_2_ was suspended in an ethanol solution and sprayed on the nickel foam. Ethanol was used for making titania suspension because it has a good affinity to the titania surface and allows for homogeneous dispersion of titania particles on the surface of nickel foam. Ethanol solution contrary to water prevents the formation of titania particles agglomerates. Such prepared composite was heat-treated at 400–600 °C under an Ar atmosphere to avoid excessive oxidation of nickel foam, which takes place above 300 °C in an oxygen atmosphere. Obtained materials were tested for NO_x_ removal under simulated environmental conditions such as UV LED irradiation of 10 W/m^2^ and relative humidity (RH) of 50%. Photocatalytic tests were carried out under a laminar flow of NO gas (1 ppm in the air) with a velocity of 1 dm^3^/min.

## 2. Results

**Figure 1:**
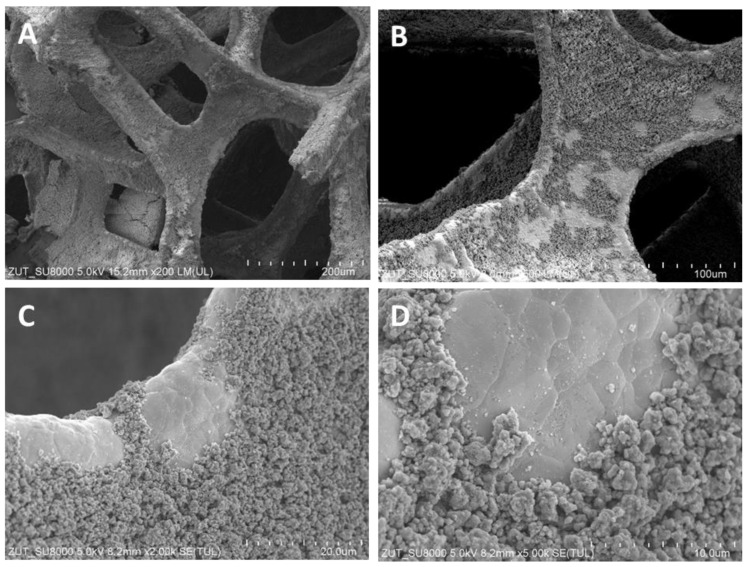
SEM images of TiO_2_/nickel foam heat-treated at 400 °C at various magnifications, (**A**) ×200; (**B**) ×500; (**C**) ×2000; (**D**) ×5000.

**Figure 2:**
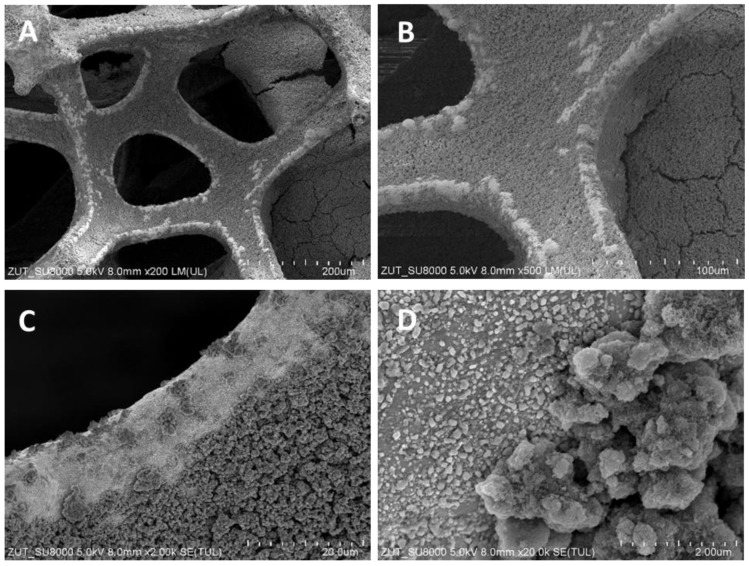
SEM images of TiO_2_/nickel foam heat-treated at 500 °C at various magnifications: (**A**) ×200; (**B**) ×500; (**C**) ×2000; (**D**) ×20,000.

**Figure 3:**
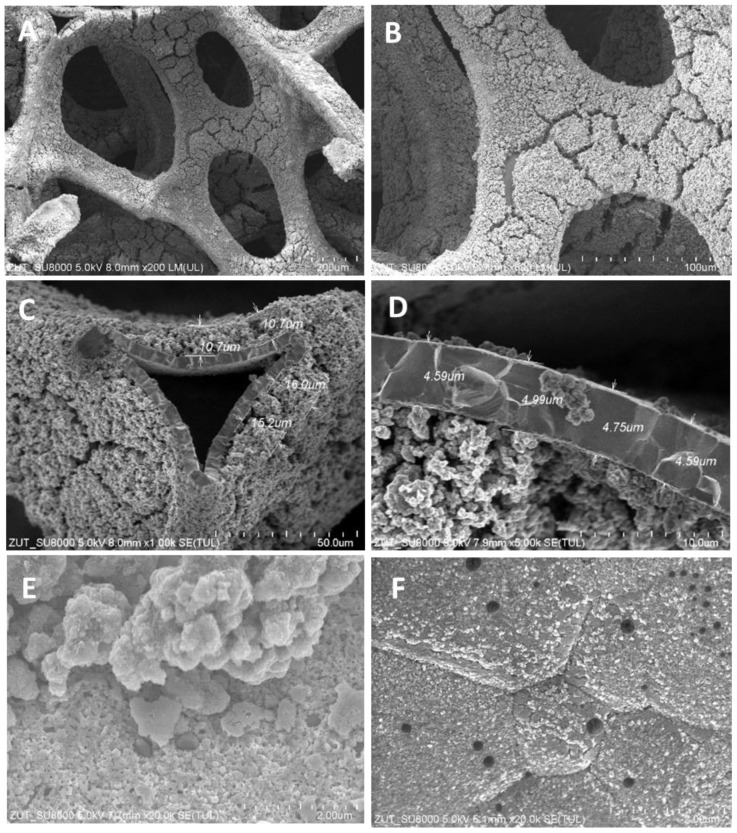
SEM images of TiO_2_/nickel foam heat-treated at 600 °C at various magnifications: (**A**) ×200; (**B**) ×500; (**C**) ×1000; (**D**) ×5000; (**E**) ×20,000; (**F**) ×20,000.

The structures of TiO_2_/nickel foam samples were investigated by SEM. In Figure 1, Figure 2 and Figure 3, selected images taken from SEM are presented.

SEM images showed various degrees of coverage of nickel foam by TiO_2_ particles. Sample heat-treated at 400 °C revealed some parts of hollow space on the edges of the nickel foam and on the flat surface as well (Figure 1). At 500 °C the coverage with TiO_2_ was somewhat higher than at 400 °C and on the bared nickel surface numerous small TiO_2_ particles were observed (Figure 2D). The sample heated at 600 °C revealed the highest coverage with TiO_2_ (Figure 3A,B). All the TiO_2_/nickel foam samples exhibited porous structures, but at 600 °C sintering of some TiO_2_ agglomerates was visible (Figure 3E). The thickness of the nickel foam matrix was around 5 µm, as shown in Figure 3D. The sample heat-treated at 600 °C had TiO_2_ coating with a layer thickness of around 10–15 µm (Figure 3C). Interestingly, some spheric nanoparticles with a size of 100–300 nm were observed on the surface of TiO_2_/nickel foam heat-treated at 600 °C (Figure 3F). Similar particles were also observed in the commercial nickel foam (Supporting Information, Figure S1). Performed EDS analyses indicated that these black spheres in Figure 3E are carbon spheres.

**Figure 4:**
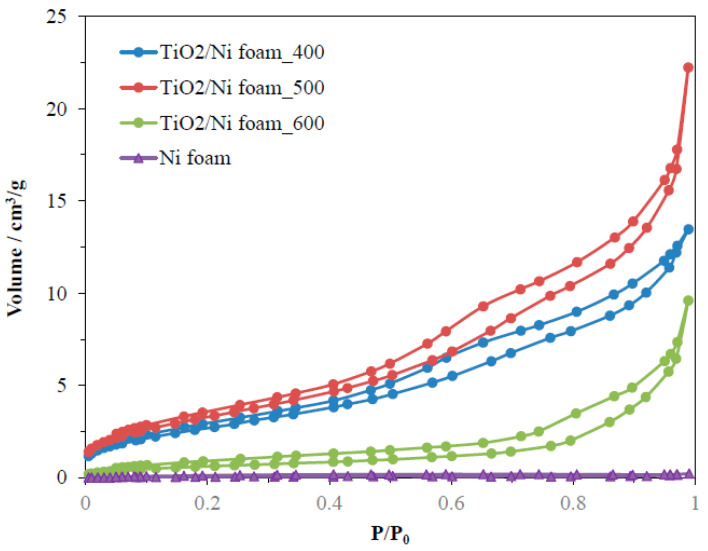
Nitrogen adsorption–desorption isotherms for Ni foam as received and coated by TiO_2_ with following heating at 400–600 °C.

The porous structure and BET surface area of TiO_2_/Ni foam composites were determined by measurements of nitrogen adsorption isotherms performed at a temperature of 77 K. Figure 4 presents the results from these measurements.

All the samples showed low adsorption of nitrogen and mesoporous characteristics. Ni foam had very large pores and was not possible to determine its porosity by this method. BET surface area determined by the BJH method and the total pore volume of samples are listed in Table 1.

The highest BET surface area and pore volume had samples heat-treated at 500 °C and the lowest values were noted for samples prepared at 600 °C. The specific surface area was related to the porous structure of TiO_2_ only, therefore the coverage of nickel foam by TiO_2_ affected the value of BET. These measurements indicated that the porosity of TiO_2_ decreases after its heating at 600 °C due to the formation of larger pores, as seen through the lowering of the adsorption curve at the low range of the relative pressure. Most likely, some agglomerates of TiO_2_ are formed and partial sintering of TiO_2_ particles takes place.

**Figure 5:**
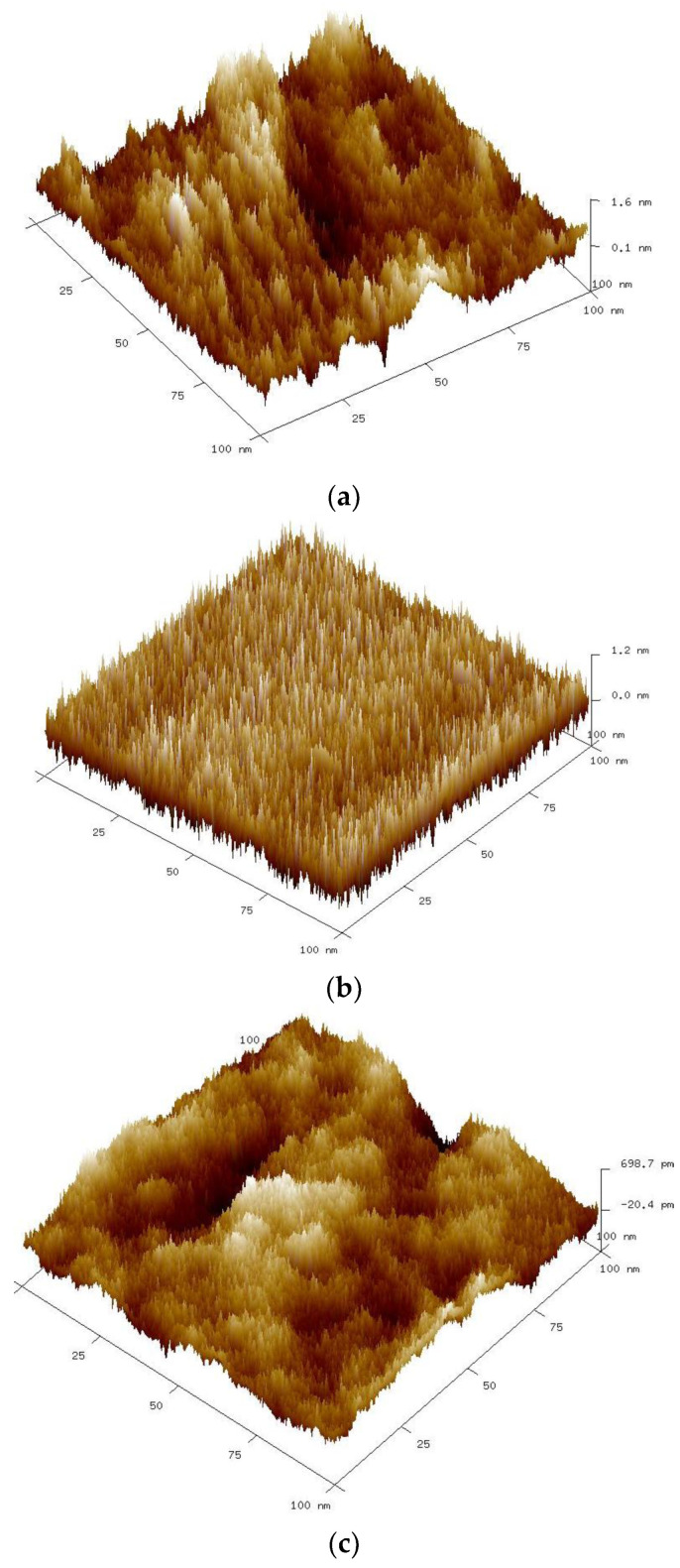
Three-dimensional AFM images of TiO_2_/Ni foams heat-treated at various temperatures: (**a**) 400, (**b**) 500, and (**c**) 600 °C.

The roughness of TiO_2_/Ni foam samples was investigated by the AFM technique. In Figure 5 are presented AFM images.

It can be seen that the temperature of the preparation affects the surface topography. The average roughness (Ra) of the nickel foams coated with TiO_2_ and heat-treated at 400, 500, and 600 °C was equal to 0.303, 0.266, and 0.155 nm, respectively. So, the average surface roughness decreases with the increasing heat-treatment temperature.

**Figure 6:**
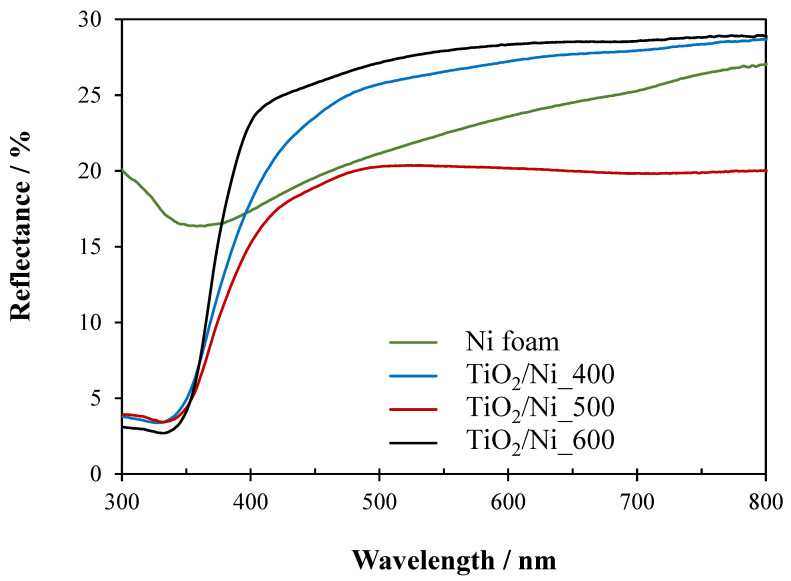
UV–Vis/DR spectra of Ni foam and TiO_2_/Ni foam samples heat-treated at various temperatures.

TiO_2_/nickel foam samples were analyzed by UV–Vis/DR spectroscopy. The obtained spectra are presented in Figure 6.

All the samples showed reflectance of 20–30% in the wavelength range of 400–800 nm. It means, that all of them absorbed a significant part of visible light. This resulted from the optical properties of the nickel foam matrix. Samples heat-treated at 600 °C revealed the highest percentage of reflectance in the range of visible light, due to the highest coverage by TiO_2_ particles.

**Figure 7:**
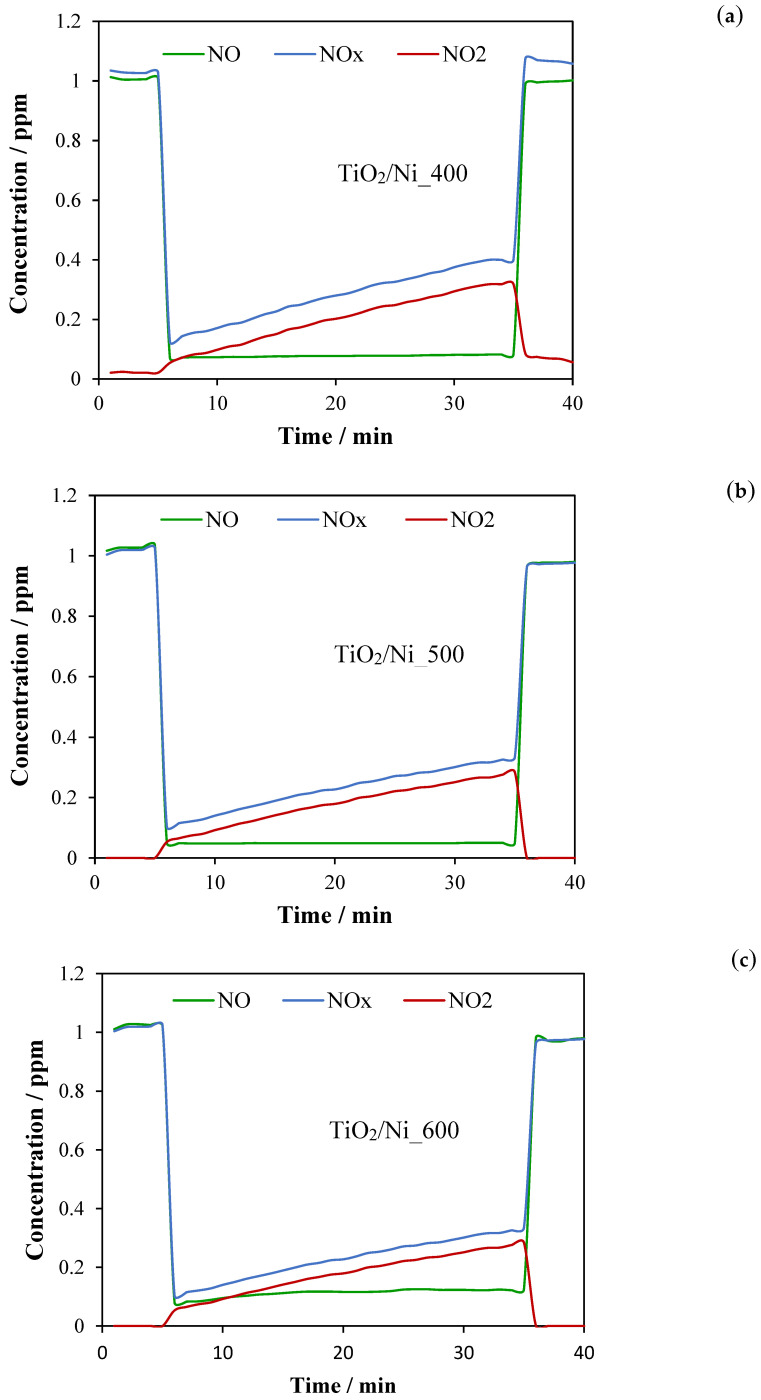
Photocatalytic conversion of NO to NO_2_ and NO_x_ abatement on TiO_2_/nickel foam heat-treated at: (**a**) 400 °C, (**b**) 500 °C, and (**c**) 600 °C; UV LED = 10 W/m^2^, RH = 50%; inlet concentration of NO = 1 ppm, flow rate = 1 dm^3^/min.

Prepared TiO_2_/nickel foam composites were tested for removal of NO_x_. Figure 7 presents results from the photocatalytic oxidation of NO to NO_2_ and the elimination of NO_x_. The measurements were performed under RH = 50% and UV LED illumination of 10 W/m^2^.

Table 2 summarizes the results from the photocatalytic NO conversion to NO_2_ and NO_x_ abatement.

These measurements indicated that NO abatement on TiO_2_/nickel foam samples was in the range of 89–95%, the highest for samples heated at 500 °C and the lowest for those heated at 600 °C. However, the highest abatement of NO_x_ was reached in the case of the sample heated at 600 °C due to the lowest emission of NO_2_ to the outlet stream of gas. These studies showed that the abatement of NO was stable in time, but with the proceeding time of the photocatalytic process, the quantity of NO_2_ in the outlet stream gradually increased. Some products of NO conversion were adsorbed on the surface of TiO_2_/nickel foam samples, but a part of them was transported to the outlet gas. These measurements showed that the sample heated at 600 °C exposed the lowest photocatalytic activity among the other samples, but the highest capacity for capturing NO oxidation products. Obtained results of NO abatement for TiO_2_/nickel foam samples were more efficient (around 10%) than those performed for TiO_2_ coating glass plate through the sol–gel method, at the same reaction conditions [19].

**Figure 8:**
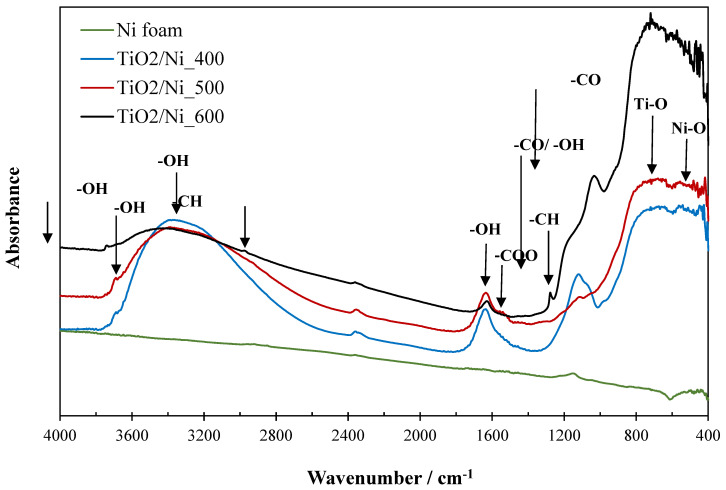
FTIR spectra of raw nickel foam and TiO_2_/nickel foam samples heat-treated at various temperatures.

The FTIR technique was utilized to analyze the chemical structure of TiO_2_/nickel foam composites before and after photocatalysis. Figure 8 presents the FTIR spectra of nickel foam as received and coated by TiO_2_ particles.

Nickel foam showed the insignificant intensity of the band at 1140 cm^−1^ assigned to the stretching vibrations of the C-O group and the broad band at the range of 600–400 cm^−1^ attributed to Ni-O bonding [15,20]. Sample heat-treated at 400 °C showed high intensity and broad band at 3700–2500 cm^−1^, characteristic of O-H vibrations in alcohols and TiO_2_ [8,21]. The other high-intensity band at the range of 1300–1011 cm^−1^ is related to the stretching vibrations of C-O and bending O-H groups in alcohols [21]. This means that there is remaining ethanol on the TiO_2_ surface in the sample heated at 400 °C. The additional bands, which are observed on FTIR spectra at 1630 and 3680 cm^−1^ are attributed to OH groups in TiO_2_ [8]. The broad band at the range of 800–600 cm^−1^ is related to Ti-O vibrations in TiO_2_ [15,20]. At 500 °C the C-O band characteristic for alcohols was almost completely reduced, instead of this -COO vibrations of the acetate group appeared at the range of 1557–1488 cm^−1^ [22]. Interestingly, the sample heated at 600 °C revealed a somewhat distinct chemical structure. The intensities of the broad band at 3700–2500 cm^−1^ and that at 1630 cm^−1^ were greatly depleted compared to the TiO_2_/nickel foam samples heated at lower temperatures. Instead of this, a small intensity band at 3740 cm^−1^ appeared as hydrogen-bounded OH groups to TiO_2_. A similar effect has been observed in our previous studies [23]. At the same time, the acetate and Ni-O groups disappeared. However, a certain reorganization of carbonaceous groups took place, and new bands at 2984 and 1270 cm^−1^ emerged related to vibrations of -CH groups, additionally, a high-intensity band at 1050 cm^−1^ appeared. It is assumed that such reorganization of carbonaceous groups was related to the appearance of some carbon spheres on the surface of TiO_2_/nickel foam heated at 600 °C. A similar chemical structure of carbon spheres was described elsewhere [24]. These carbon spheres can be partly observed on the raw nickel foam as well [25].

**Figure 9:**
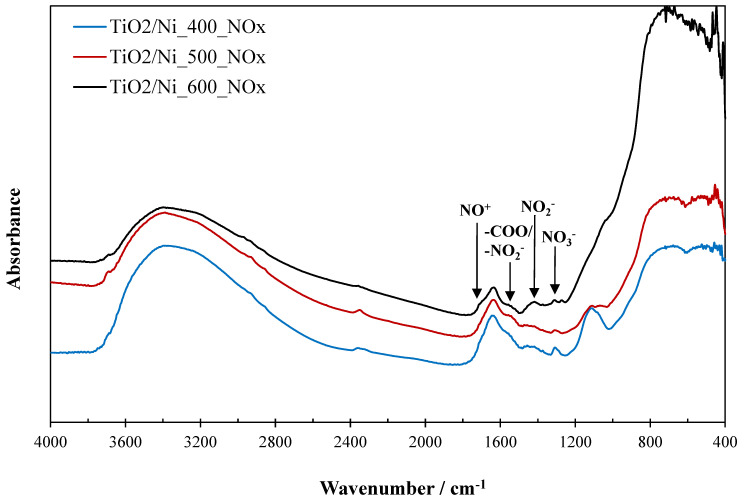
FTIR spectra of TiO_2_/nickel foam samples after photocatalytic process.

FTIR spectra of TiO_2_/nickel foam samples were measured after the photocatalytic process to analyze the adsorption of some nitrogen species on the titania surface. Results from the measurements are presented in Figure 9.

Some nitrogen species were observed on the TiO_2_/nickel foam samples after their exposition to photocatalytic conversion of NO. NO_3_^−^ species were detected on FTIR spectra at a wavenumber of around 1300 cm^−1^ [8]. They were higher intensity for samples heated at lower temperatures (Figure 9). However, opposite to this, NO_2_^−^ species were accumulated more intensively on the sample heated at 600 °C (bands at 1480–1360 cm^−1^ and 1540 cm^−1^) [8,15] than on those prepared at lower temperatures. NO^+^ species (band at around 1700 cm^−1^) [15] was clearly visible for the sample heated at 600 °C.

The chemical structure of TiO_2_/nickel foam samples was also analyzed by the XPS method. Table 3 lists the overall compositions of TiO_2_/nickel foam samples.

The highest intensity of the Ti2p signal was obtained for the sample heated at 600 °C, due to the highest loading of TiO_2_ on its surface, and at the same time, the Ni2p_3/2_ signal was shielding. Carbonaceous species were present in all the samples, but their quantity decreased with the increase in the temperature of heating. All these samples contained insignificant content of potassium, around 1 at.%, which was probably a residue in titania raw material.

**Figure 10:**
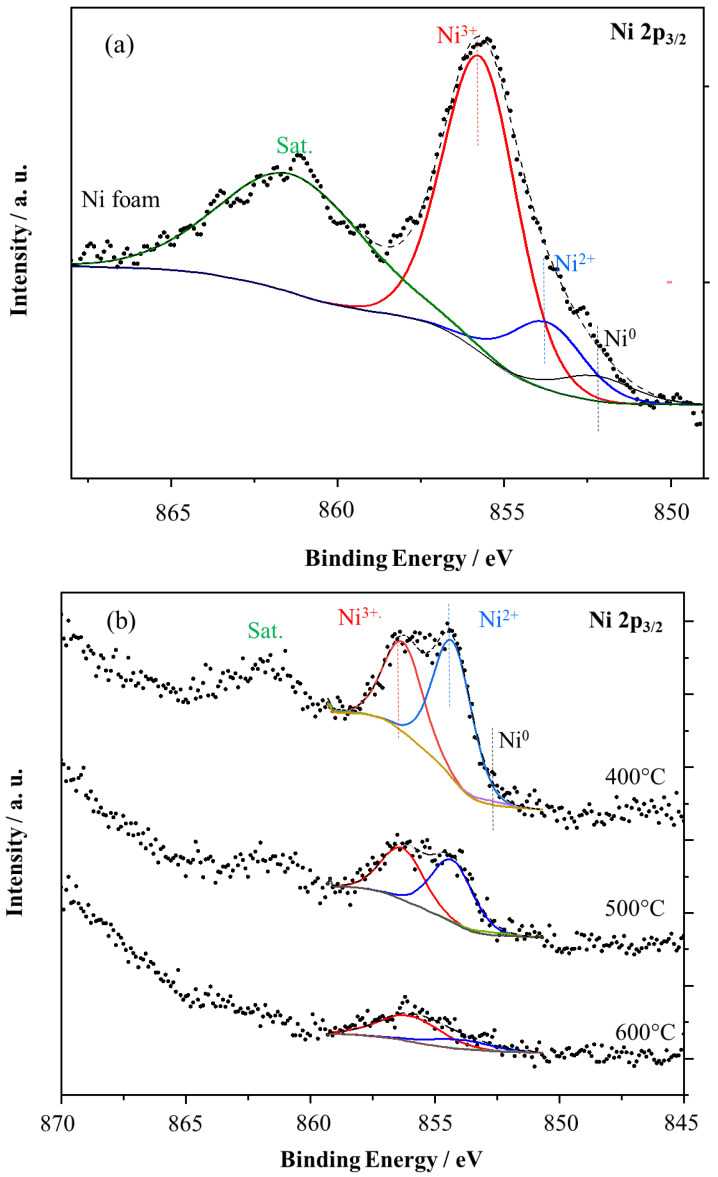

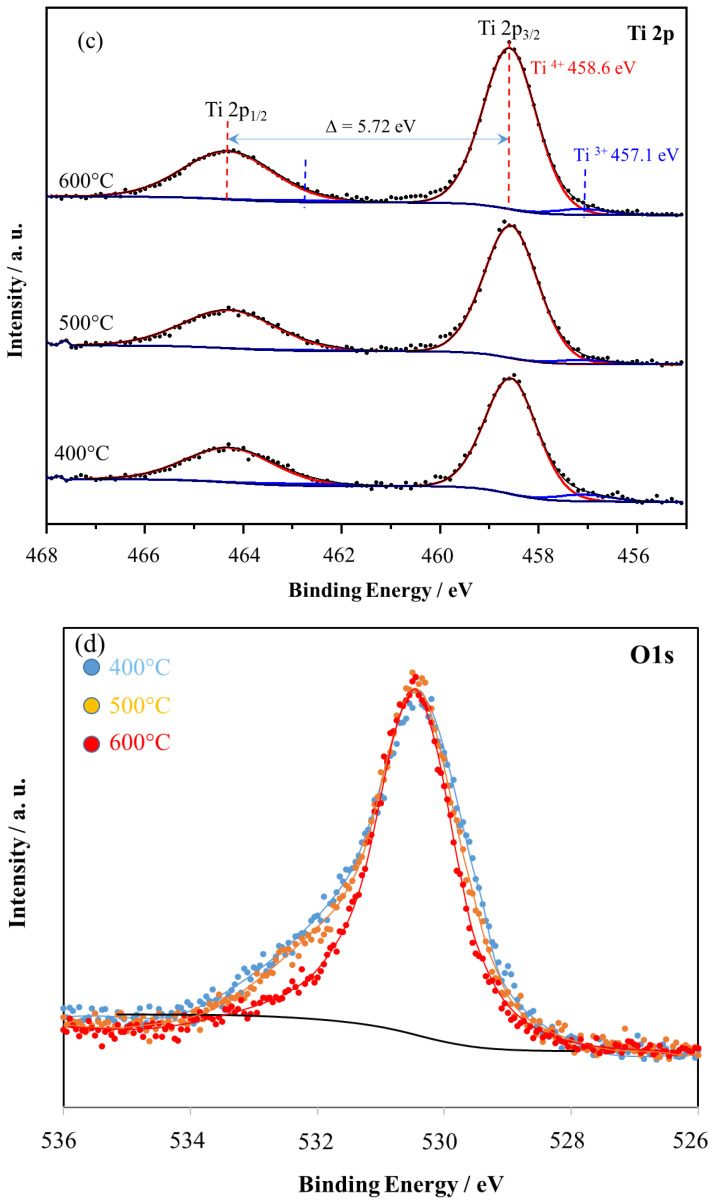
XPS spectra for (**a**) Ni2p of nickel foam and (**b**) Ni2p, (**c**) Ti2p, (**d**) O1s of TiO_2_/nickel foam samples heated at 400–600 °C.

Figure 10a–d presents the XPS spectra for Ni2p, Ti2p, and O1s species of TiO_2_/nickel foam samples.

Ni2p_3/2_ signal in nickel foam (Figure 10a) can be deconvoluted to Ni^0^, Ni^2+^, Ni^3+^ (852.3, 853.8, and 855.8 eV, respectively [15]. The signal of Ni^0^ is negligible. The metallic Ni is present in the nickel foam, but the XPS sampling depth is c.a. 1 nm, therefore, mostly the nickel oxide species are observed. In the case of TiO_2_/nickel foam samples Ni2p_3/2_ signal was attenuated by TiO_2_, and, therefore was lower intensity than in nickel foam itself, and intensity decreased with the amount of loading TiO_2_ (Figure 10b). However, the composition of nickel oxides was changed in the sample heated at 600 °C, the only Ni^3+^ signal was observed instead of two Ni^3+^ and Ni^2+^. Such a phenomenon can be related to the diffusion of NiO to TiO_2_, as we described above. The measurements of the Ti2p signal (Figure 10c) indicated two peaks at 458.6 and 464.6 eV as a result of spin–orbit splitting, and these could be assigned to Ti^4+^. Deconvolution of Ti2p_3/2_ allowed us to identify a small peak at lower binding energy such as 457.1 eV, attributed to Ti^3+^. The intensity of the Ti^3+^ peak was insignificant, somewhat higher in samples prepared at 400 and 600 °C than in those heated at 500 °C. The presence of Ti^3+^ can be related to some defects in titania crystals, at 400 °C caused by not complete crystallization, but at 600 °C by incorporation of some NiO species.

The deconvolution of the O1s spectrum was not performed due to the presence of many components, which should be taken into account. This spectrum is expected to be the convolution of oxygen signals from TiO_2_, NiO, Ni_2_O_3_, COOH, CO, COH, Ti-OH, Ni-OH, and others. Moreover, the investigated material has a sandwich-like structure with TiO_2_ on the top and NiO beneath. This causes attenuation effects which makes the problem even more complicated. Therefore, this problem cannot be solved unambiguously by standard fitting procedure. It can be solved with many arbitrarily taken constraints, but the final result will be as good as good will be assumption. Q. Zeng et al. performed deconvolution of such O1s spectrum with simplified assumptions, however, no consistent results were obtained [15]. Therefore, the O1s signal was presented as overlapped normalized spectra. There is observed a disappearance of the shoulder for the sample heated at 600 °C, probably due to the dehydration of TiO_2_. In this region, there are expected both species, Ti-OH and Ni-OH, at 531.1 and 532.4 eV, respectively [15]. These insights cover those obtained from FTIR measurements.

**Figure 11:**
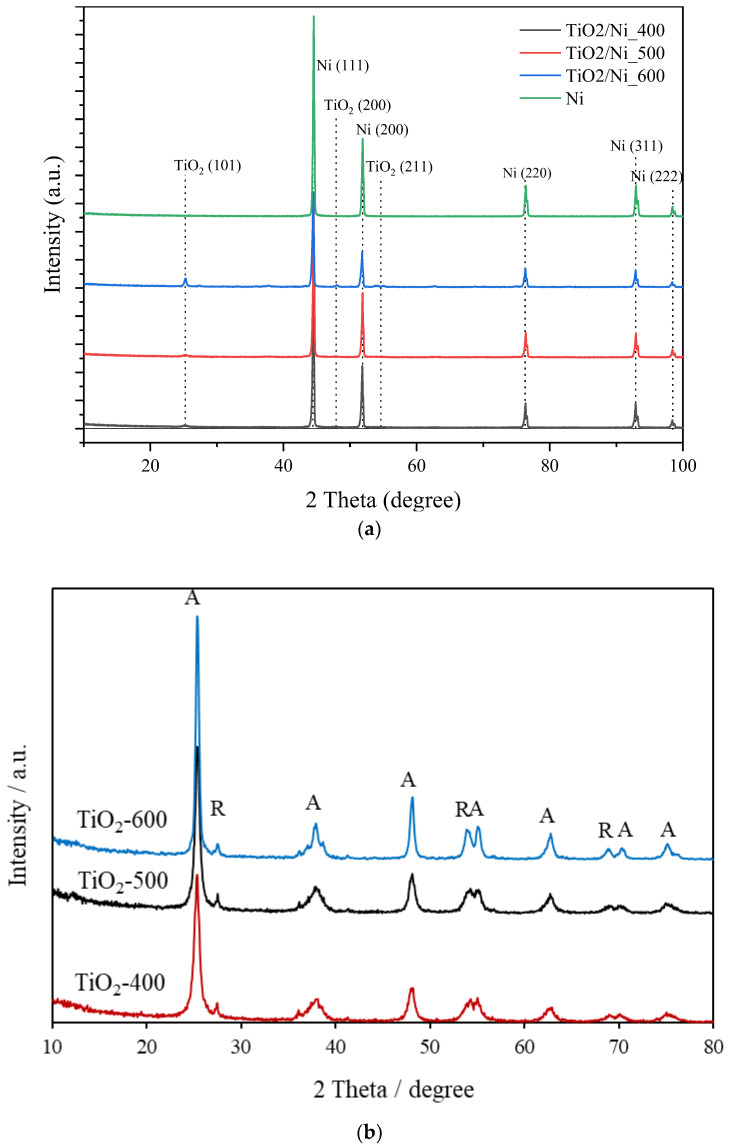
XRD patterns of (**a**) nickel foam alone and loaded with TiO_2_ and (**b**) TiO_2_ powders, heated at 400–600 °C; (A—anatase; R—rutile).

The phase compositions of TiO_2_/nickel foam composites and TiO_2_ itself were analyzed by XRD measurements. Figure 11 presents obtained XRD patterns.

XRD measurements of nickel foam showed reflections of metallic nickel phase without any nickel oxide structures. TiO_2_ of the anatase phase was visible for the sample heat-treated at 600 °C. which contained the highest quantity of loaded TiO_2_ [15]. Although XPS measurements confirmed the presence of nickel oxides on nickel foam, XRD patterns showed only the metallic phase of nickel. Most likely, the layer of nickel oxides was very thin. The phase composition of TiO_2_ was unchanged during heating at 400–600 °C, it consisted mainly of anatase and contained a small amount of rutile. More sharp and intensive reflexes of anatase for the sample heated at 600 °C (Figure 11b) indicate its more crystalline structure by comparison with other samples.

## 3. Materials and Methods

### 3.1. Preparation of TiO_2_/Nickel Foam Composites

Nickel foam sheets (China) with purity of 99.8% were used as the support for TiO_2_. The parameters of nickel foam declared by a producer were as follows: thickness of 1.5 mm, porosity of 95–97%, and surface density of 300 g/m^2^. As a source of TiO_2_, nanocrystalline anatase was used with an average crystallite size of around 15 nm and BET surface area of 215 m^2^/g. This material was prepared by the hydrothermal treatment of amorphous titania pulp obtained from the industrial production of titania white in Police Chemical Factory (Grupa Azoty, S.A., Police, Poland). The hydrothermal treatment of titania aqueous suspension proceeded at 150 °C under pressure of 7.4 bar for 1 h. Details of the preparation method were published in our previous paper [26].

TiO_2_ was suspended in ethanol (0.5 g TiO_2_ per 100 mL of ethanol) and spread out on the nickel foam using aerograph. Prior to spreading, suspension was sonicated for 15 min in a sonic bath to achieve a good dispersion of titania powder in the liquid. In total 1.0 g of TiO_2_ was used for loading on nickel foam sheet of size 5 × 25 cm. However, it was estimated by the weight method, that amount of deposited TiO_2_ on the nickel foam was around 0.5–0.6 g due to some losses of TiO_2_ during spraying process. In total, 3 nickel foam sheets with loaded TiO_2_ were prepared, dried, and then heat-treated in a tube furnace at 400 °C, 500 °C, and 600 °C under flow of Ar gas. The thermal ramp-up was 10 °C/min and the samples were kept at final temperature for 2 h. Argon atmosphere was used instead of air to prevent oxidation of nickel foam. Our previous studies revealed that oxidized nickel foam was disadvantageous for the photocatalytic decomposition of acetaldehyde in air under UV LED irradiation [25].

### 3.2. Analytical Techniques

XRD measurements of TiO_2_/Ni foams were performed using the diffractometer (PANanalytical, Almelo, The Netherlands) with a Cu X-ray source, λ = 0.154439 nm. Measurements were conducted in the 2θ range of 10–100° with a step size of 0.013. The applied parameters of Cu lamp were: 35 kV and 30 mA. The same conditions were used for measurements of powdered TiO_2_ (without nickel foam support), heated at 400–600 °C in Ar.

SEM/EDS images were obtained using a field emission scanning electron microscope with high resolution (UHR FE-SEM, Hitachi SU8020, Tokyo, Japan).

The chemical structure of TiO_2_/nickel foam samples was determined by both, FTIR and X-ray photoelectron spectroscopy (XPS) measurements. FTIR spectra were recorded for TiO_2_/nickel foam samples before and after photocatalytic tests of NO_x_ abatement. All the FTIR spectra were received from JASCO 4200 FTIR spectrometer (Jasco Inc., Tokyo, Japan) with a resolution of 4 cm^−1^. XPS measurements were conducted using a commercial multipurpose ultra-high vacuum (UHV) surface analysis system (PREVAC, Rogow, Poland). A nonmonochromatic XPS source and a kinetic electron energy analyzer (SES 2002; Scienta, Uppsala, Sweden) were used. The spectrometer was calibrated using the Ag 3d5/2 transition. The XPS analysis utilized Mg Kα (h = 1253.6 eV) radiation as the excitation source. UV–Vis/DRS spectroscopy was applied to analyze the optical properties of TiO_2_/nickel foam samples. The measurements were performed in V-650 spectrometer from Jasco (Tokyo, Japan). The spectra were recorded in the range of 200–800 nm with the scanning speed of 1 nm/s. As a reference, BaSO_4_ was used.

The porous structure of TiO_2_/nickel foam samples was determined by measurements of nitrogen adsorption isotherms at low temperatures (77 K) using QUADRASORB Si analyzer (Quantachrome, Boynton Beach, FL, USA). Prior measurements samples were heated in a drier overnight and then were degassed at 200 °C for 12 h under high vacuum using MasterPrep degasser by Quantachrome, Boynton Beach, FL, USA.

The surface roughness of TiO_2_/Ni foam composites was analyzed by Atomic Force Microscopy (AFM) using Bruker NanoScope V Multimode 8 (Billerica, MA, USA). Prior to measurement, the tested samples were attached to a steel disc using double-sided tape. Measurements were taken in ScanAsyst mode using a silicon nitride probe. The applied scan rate was 1 Hz and the area scanned 100 nm × 100 nm. Three-dimensional AFM images and average roughness (Ra) values were obtained using NanoScope Analysis (v1.40) software.

### 3.3. Photocatalytic Removal of NOx

The photocatalytic activities of TiO_2_/nickel foam samples were studied for Nox removal in a continuous flow reactor under UV LED illumination (λ = 365 nm) with radiation intensity of 10 W/m^2^. The radiation intensity was controlled by a radiometer (UV Light Meter UV510, Extech Instruments, Poissy, France). The photocatalytic system was set up according to the ISO standard 22197-1:2007 [27]. It was equipped with Nox analyzer (Heated Chemiluminescence Nitrogen Oxide Analyzer Model ENVIRONNEMENT S.A. AC32M). Details of this photocatalytic system were described in the other paper [19]. Process was carried out at a temperature of 28 ± 1 °C and relative humidity (RH) of 50 ± 1%. The initial concentration of NO in air was 1 ppm. Gas was flowing through the reactor with velocity of 1 dm^3^/min, above the TiO_2_/nickel foam sample. The laminar flow was maintained. At the beginning, process was carried out in dark, and then after 5 min UV lamp was turned on. NO molecules were oxidized to NO_2_ and adsorbed on the titania surface. Monitored results were expressed as the ability of the TiO_2_/nickel foam to abate the total NOx concentration from the air stream during 30 min of UV irradiation. After that time UV lamp was turned off and the initial level of NO gas concentration was achieved. NO_2_ is usually produced during the photocatalytic oxidation of NO [28] therefore NO_2_ concentration was also recorded. The photocatalytic activity of TiO_2_/nickel foam was evaluated by abatement of both concentrations, NO and NOx according to the following formulas:

(1) $$NO\;abatement=\frac{{\left[NO\right]}_{in}-{\left[NO\right]}_{out}}{{\left[NO\right]}_{in}}\;\cdot 100\%$$

(2) $${NO}_{x}\;abatement=\frac{{\left[{NO}_{x}\right]}_{in}-{\left[{NO}_{x}\right]}_{out}}{{\left[{NO}_{x}\right]}_{in}}\;\cdot 100\%$$

The percentage of NO_2_ present in the outlet stream in relation to abatement of initial NO concentration was calculated as follows:

(3) $${NO}_{2}=\frac{{\left[{NO}_{2}\right]}_{out}}{{\left[NO\right]}_{in}-{\left[NO\right]}_{out}}\;\cdot 100\%$$

## 4. Discussion

Loading of TiO_2_ on the nickel foam from the ethanol suspension and heat treatment at 600 °C conducted to the sintering of TiO_2_ with the support. diffusion of NiO to the TiO_2_ occurred, as it was deduced from XPS measurements. As a result, most likely, the NiTiO_3_ phase was formed. NiO starts to crystalize at 600 °C and then the formation of NiTiO_3_ is possible, as reported elsewhere [29]. A similar effect was observed by other researchers, who calcined TiO_2_/nickel foam at 600 °C [15]. This phenomenon did not occur at lower temperatures. The sample heated at 600 °C had a less porous structure than the other samples and some carbon spheres emerged on the titania surface, which probably originated from some nickel foam impurities. Ethanol used for spreading out TiO_2_ particles on nickel foam remained on the titania surface, as evidenced by FTIR measurements, however, it was gradually decomposed when samples were heated at higher temperatures. High hydroxylation of the titania surface observed for the sample heated at 400 °C facilitated oxidation of NO to NO_3_^−^ ions and their adsorption on the titania surface whereas for low hydroxylated TiO_2_, adsorption of NO_2_^−^ and NO^+^ ions was dominated. It is known from the literature [6] that NO_3_^−^ at the moisture conditions and UV light undergoes photolysis to NO_2_. Therefore, samples that revealed dominant adsorption of NO_3_^−^ ions showed also higher selectivity of NO conversion to NO_2_. Generally, photocatalytic abatement of NO_x_ was the most efficient for TiO_2_/nickel foam heat-treated at 600 °C, although the sample obtained at 500 °C showed the highest oxidation of NO to NO_2_. Photocatalytic removal of NO_x_ should cover the transformation of all the nitrogen oxide species towards safe compounds to avoid the generation of toxic NO_2_ gases in the atmosphere. Some researchers reported that in the absence of water, adsorbed NO_3_^−^ was mainly converted to adsorbed NO_2_ and N_red_. Whereas in the presence of moisture, adsorbed NO_3_^−^ was converted to gas-phase products [6]. The other researchers proved that photogenerated electrons captured by NO drive the transformation of NO_3_^−^ under light irradiation via the pathway: NO_3_^−^ + NO^−^ → 2NO_2_^−^. Additionally, although photogenerated holes and hydroxyl radicals could oxidize NO into NO_3_^−^, the rate of production of NO_3_^−^ is much slower than that of the photochemical transformation of NO_3_^−^ by NO^−^ [30]. Therefore, adsorbed NO on the TiO_2_ surface can participate in the formation of secondary pollutants in air through the photocatalytic transformation of NO_3_^−^. From this point of view, the adsorption of NO_3_^−^ species on TiO_2_ is adverse for effective NO_x_ abatement as well as high concentrations of moisture in the atmosphere [15]. The other researchers indicated that induced under UV irradiation O_2_^−^ radicals easily reacted with NO and participated in its photocatalytic oxidation [12]. The amount of O_2_^−^ radicals formed upon UV irradiation depended on the calcination temperature of TiO_2_ and was higher for lower temperatures of heating, such as 300 °C. This could be related to the mobility of free electrons, which is higher for amorphous and more defective TiO_2_. They also observed the interaction of adsorbed NO_2_ with the Ti-OH group as the following disproportion: 3NO_2_ + 2OH^−^ → 2NO_3_^−^ + NO + H_2_O [12], which is consistent with our results. TiO_2_/nickel foam samples heat-treated at 400 and 600 °C had somewhat lower photocatalytic activity towards NO oxidation than that prepared at 500 °C due to the possible electron traps by carbon impurities present on the TiO_2_ surface. Desorption of carbon species from the titania surface at 500 °C could cause the formation of oxygen vacancies and titania reduction. In the case of this sample, some adsorbed acetate groups were present on the titania surface, which is typical for the reduced TiO_2_. Under UV irradiation and moisture, the hydroxyl groups are adsorbed on the titania vacancy sites, which conducts to formation of hydroxyl radicals, which take place in NO oxidation. Therefore, the sample obtained at 500 °C revealed a high transformation of NO to NO_2_. Although XPS measurements revealed an insignificant increase in Ti^3+^ centers on the TiO_2_/Ni foam heat-treated at 600 °C, this sample showed lower activity for NO oxidation than the others. In this case, NO species were transported to the titania oxygen vacancy sites and an ilmenite structure was formed (NiTiO_3_). This structure did not enhance the photocatalytic activity of titania.

## 5. Conclusions

Photocatalytic activity towards abatement of NO_x_ in the air was tested for TO_2_ loaded on the nickel foam and heat-treated at 400–600 °C in an argon atmosphere. TiO_2_ was mounted on the nickel foam from the suspension of TiO_2_ in ethanol. Application of ethanol allowed to obtain good dispersion of titania particles. However, the remaining alcohol species on the titania surface at low temperatures of heating such as 400 °C insignificantly decreased its photocatalytic activity. At 500 °C desorbed carbonaceous species from the titania surface most likely conducted to its reduction and formation of oxygen vacancies. Prepared in this temperature composite revealed the highest photocatalytic activity towards the oxidation of NO to NO_2_. Composites prepared at 400 and 500 °C showed high adsorption of NO_3_^−^ on the titania surface contrary to that one prepared at 600 °C, which revealed high adsorption of NO_2_^−^. High hydroxylation of the titania surface conducts to transformation of NO to NO_3_^−^, but at the conditions of UV and moisture, these adsorbed ions decompose to NO_2_, which results in lower photocatalytic NO_x_ abatement. Therefore, a less hydroxylated surface of TiO_2_ seems to be more favorable for NO_x_ abatement, because facilitates the adsorption of NO_2_ species, which are more stable and do not desorb to the atmosphere. Heating of TiO_2_ loaded on nickel foam at 600 °C conducts to formation of NiTiO_3_ structure, which partly decreases the photocatalytic activity of TiO_2_. These studies revealed that higher temperatures of heating enhanced the adhesion of TiO_2_ to nickel foam and caused higher coverage with TiO_2_ having low hydroxylation of surface, which appeared to be beneficial for NO_x_ abatement. However, the formation of NiTiO_3_ was detrimental and should be avoided during the preparation of such a composite. The carbon impurities of nickel foam could affect the photocatalytic activity of prepared TiO_2_/nickel foam composites, therefore purified nickel foam should be used for preparation.

## Figures and Tables

**Table 1:** BET surface area and total pore volume of TiO_2_/Ni foam composites.

Sample	BET Surface Area (m^2^/g)	Total Pore Volume (cm^3^/g)
TiO_2_/Ni foam_400	10	0.021
TiO_2_/Ni foam_500	13	0.034
TiO_2_/Ni foam_600	2.4	0.015

**Table 2:** Abatement of NO and its conversion to NO_2_ in the photocatalytic process after 30 min of UV illumination conducted on TiO_2_/nickel foam samples heated at 400–600 °C.

Sample	NO Abatement (%)	Selectivity to NO_2_ (%)	NO_x_ Abatement (%)
TiO_2_/Ni foam_400	92	34	62
TiO_2_/Ni foam_500	95	28	68
TiO_2_/Ni foam_600	89	13	76

**Table 3:** Surface composition of TiO_2_/nickel foam heat-treated at 400–600 °C (from XPS analyses).

HTT	O1s	C1s	K2p	Ni2p_3/2_	K2p
(°C)	(% at.)				
400	65.4	13.4	0.9	6.4	13.9
500	65.5	11.2	0.9	3.2	19.2
600	64.1	9.7	1.3	1.3	23.5

## Data Availability

The data presented in this study are available on request from the corresponding author.

## Supplementary Materials

The following supporting information can be downloaded at: https://www.mdpi.com/article/10.3390/molecules29081766/s1.
