# Supplementary figures and images for: Application of TiO2 Supported on Nickel Foam for Limitation of NOx in the Air via Photocatalytic Processes
Source: Molecules. 2024 Apr 12;29(8):1766. doi: 10.3390/molecules29081766 (PMC11052280; doi:10.3390/molecules29081766)

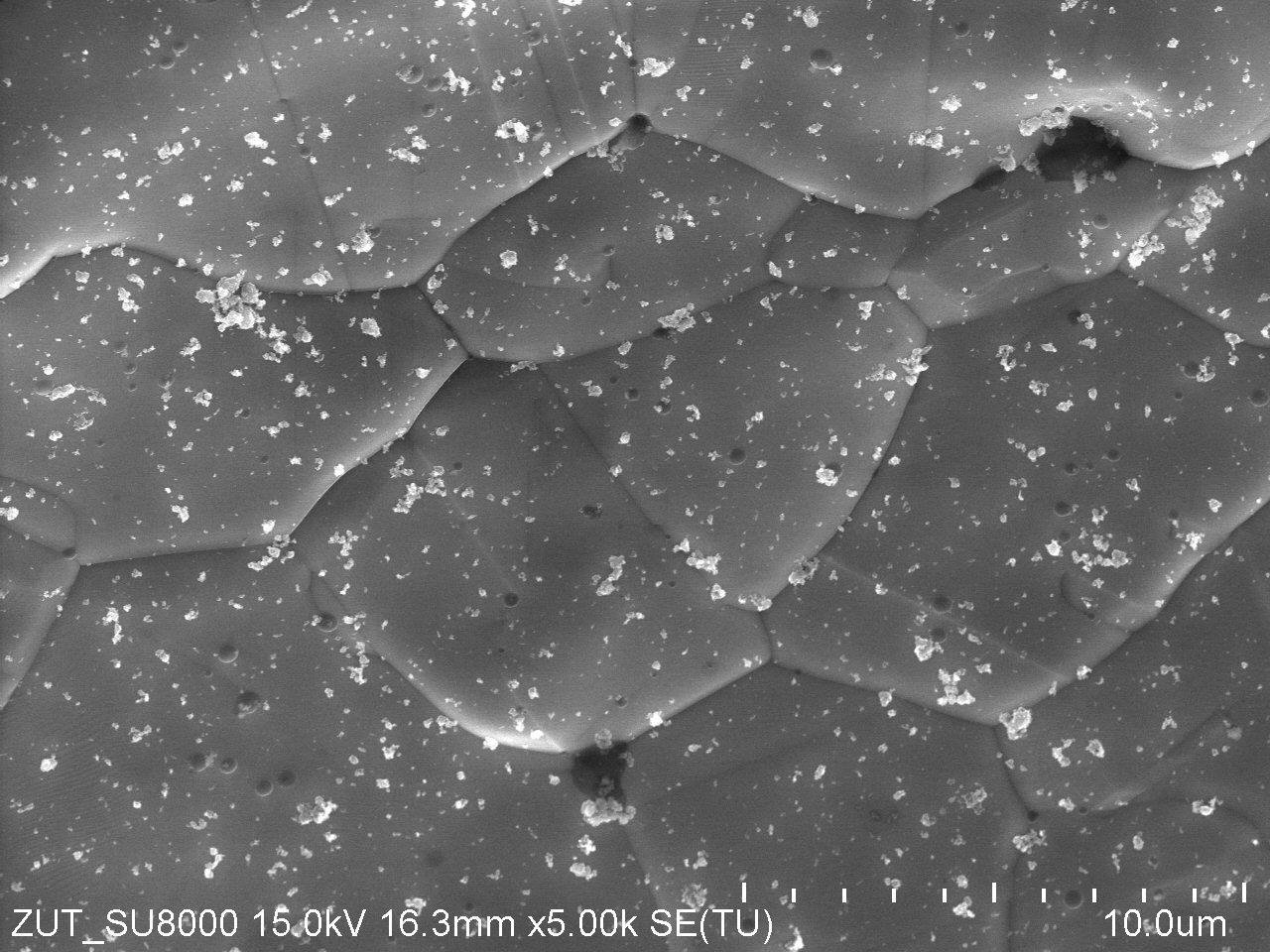

Supplement: Supplementary file 1 [file molecules-29-01766-s001.zip › molecules-2938498-supplementary.jpg]
